# Supplementary material for: Extreme genetic signatures of local adaptation during Lotus japonicus colonization of Japan
Source: Nat Commun. 2020 Jan 14;11:253. doi: 10.1038/s41467-019-14213-y (PMC6959357; doi:10.1038/s41467-019-14213-y)
Supplement: Supplementary file 1 — Supplementary information [file 41467_2019_14213_MOESM1_ESM.pdf]

Extreme genetic signatures of local adaptation during *Lotus japonicus* colonization of Japan

Shah *et al.*

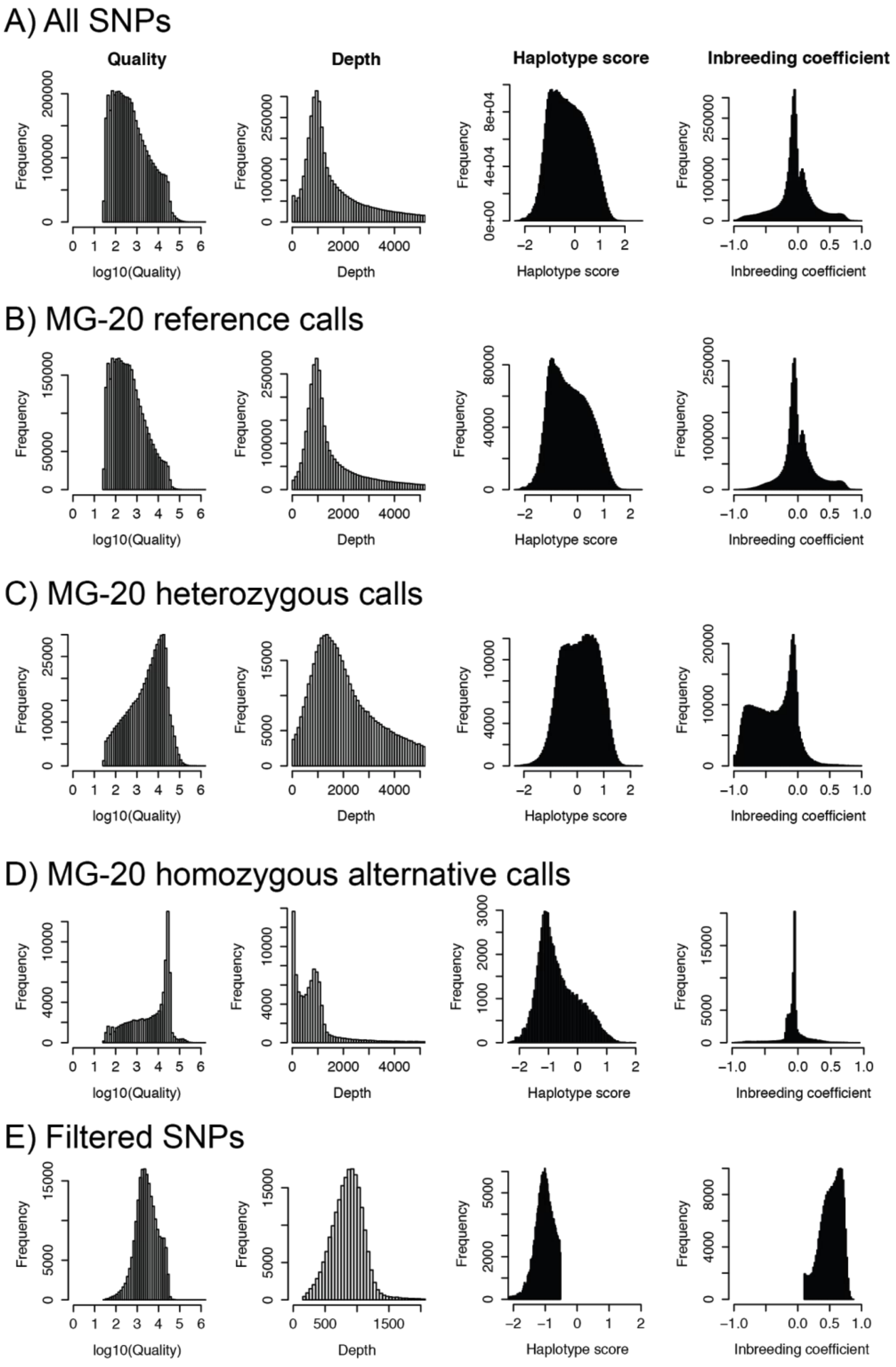

Supplementary Figure 1. SNP property summary. Histograms for total sequencing depth, alternative allele call quality, haplotype score and inbreeding coefficients as calculated by the GATK pipeline are shown for the SNPs detected on anchored contigs. **A**) All positions. **B**)

Positions with an MG20 homozygous reference call. **C**) Positions with an MG20 heterozygous call. **D**) Positions with an MG20 homozygous alternative allele call. **E**) Positions remaining after filtering requiring an MG-20 reference call, total depth > 150, Quality > 30, Haplotype score <-0.3, Inbreeding coefficient >0.1, no more than 50% missing data, and a minor allele frequency of at least 5%. The haplotype score indicates the consistency of the site with two (and only two) segregating haplotypes. Higher scores are indicative of regions with low quality alignments, which can often result in false positive SNP and indel calls. The inbreeding coefficient is the result of a likelihood-based test for the level of inbreeding. Source data are provided as a Source Data file.

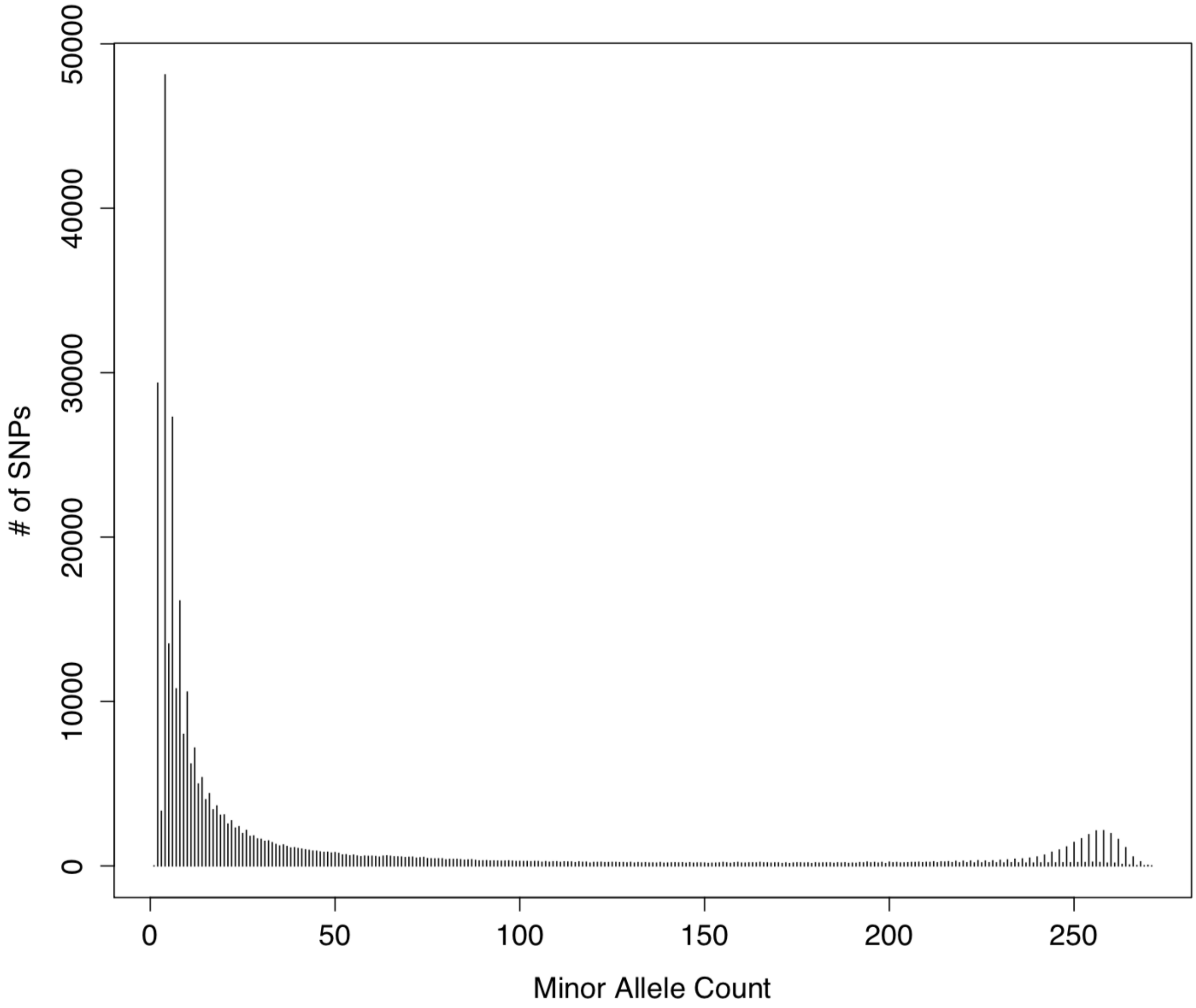

Supplementary Figure 2. Site frequency spectrum. Non-reference allele count of the SNPs in the non-repetitive regions of the genome.

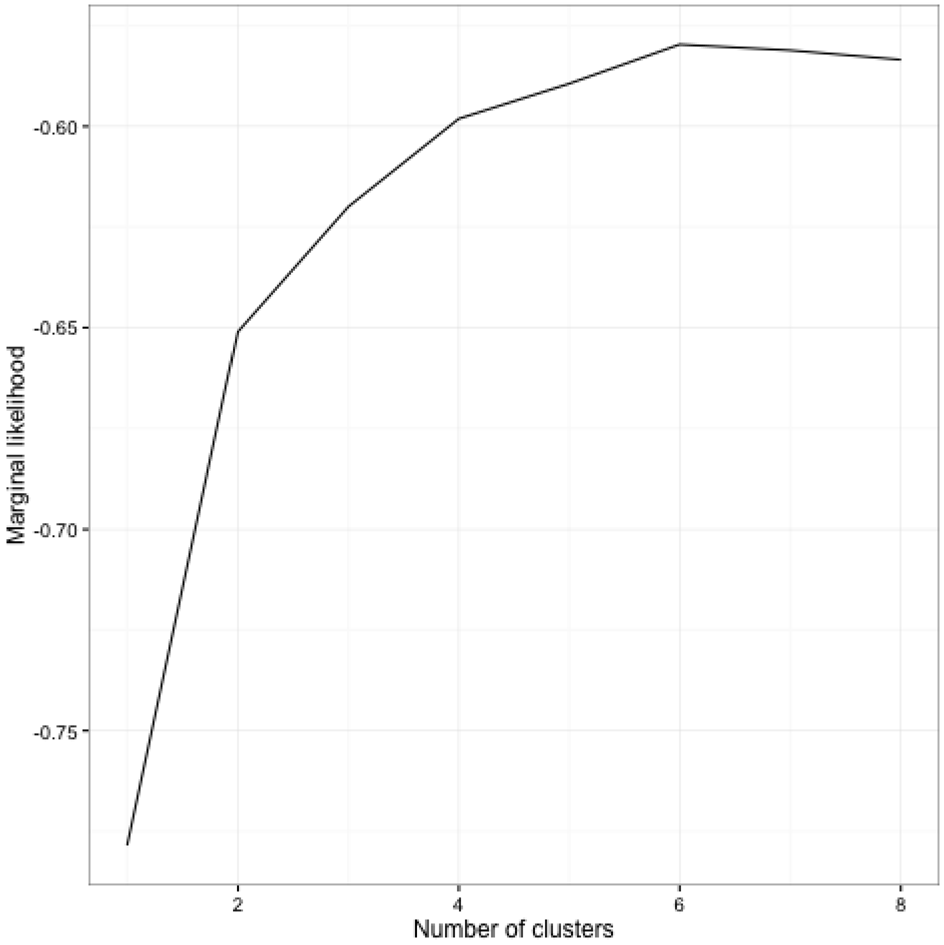

Supplementary Figure 3. Estimating the number of *Lotus* subpopulations. Marginal likelihoods as a function of the number of subpopulations (*K*) in the fastSTRUCTURE analysis.

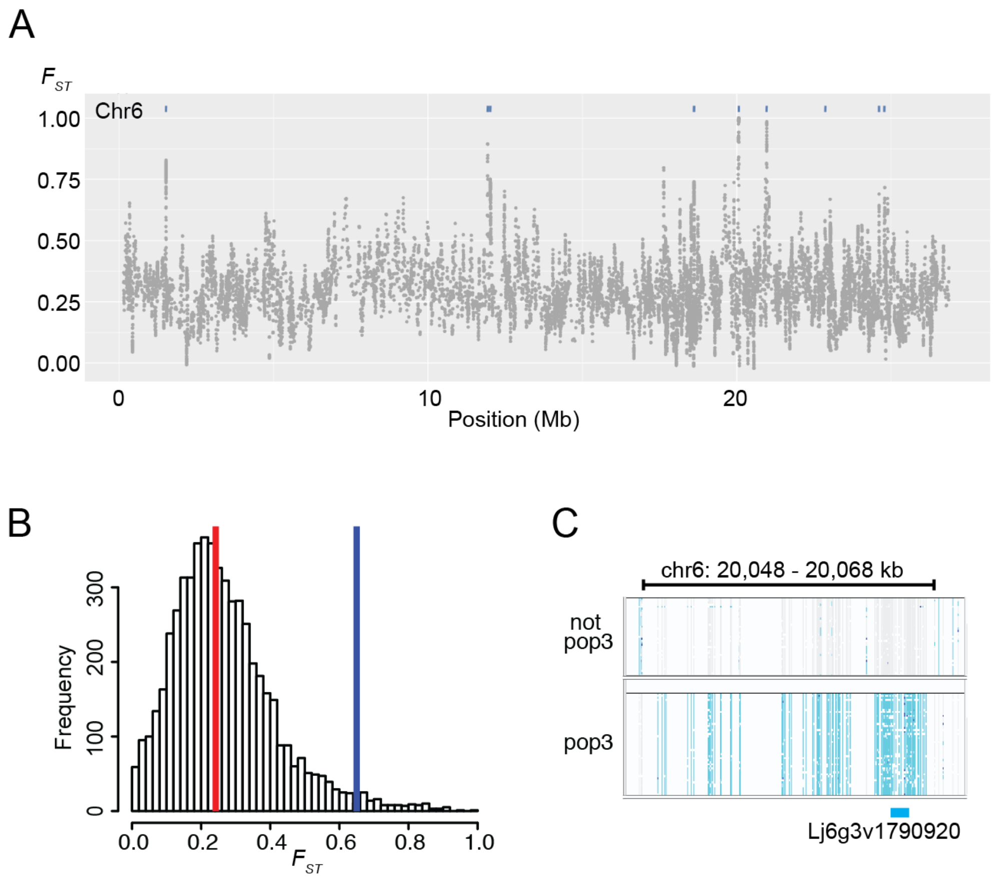

Supplementary Figure 4. Population 3 differentiation. A) Fixation index (F_{ST}) for accessions with more than 99% pop3 membership versus accessions with less than 0.1% pop3 membership. Each grey dot represents the average F_{ST} for 10 SNPs. Blue bars indicate the positions of genes with at least four informative SNPs and an average F_{ST} above 0.65. **B**) Histogram of average F_{ST} per gene. A total of 5,612 genes with at least 4 informative SNPs were included in the analysis. The vertical lines indicate the median F_{ST} (red) and an F_{ST} of 0.65 (blue). **C**) Genotype visualization for accessions with full or no pop3 membership in the genomic region containing the gene *Lj6g3v1790920*, which had the highest average F_{ST} score. Genotypes are indicated by colors: homozygous reference (grey), homozygous alternative (light blue), heterozygous (dark blue), and no call (white).

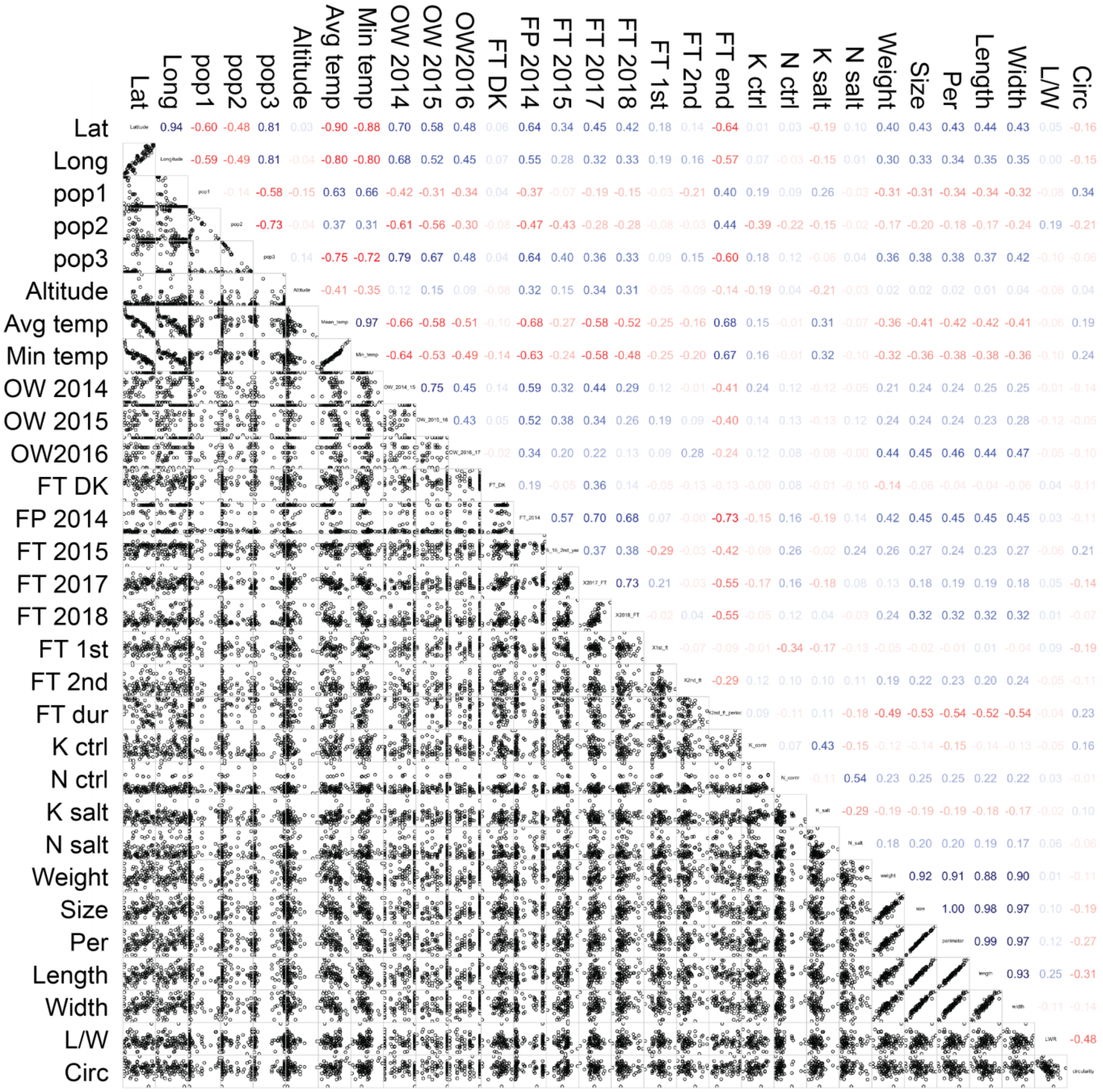

Supplementary Figure 5. Phenotype correlations. Pairwise correlations between sets of phenotype and environmental data. Scatter plots are shown below and Pearson correlation coefficients are listed above the diagonal. Positive (blue) and negative (red) correlations are indicated. Lat: latitude. Long: longitude. pop1-3: population membership based on fastSTRUCTURE analysis (see **Figure 2** and **Supplementary Data 1**). Lat, Long, Altitude, Average (Avg) and Minimum Temperature (temp) refer to accession collection sites. OW:

overwintering, the fraction of plants surviving winter. FP 2014: Proportion of plants flowering at the Tohoku field site in 2014. FT: flowering time. FT DK: days to flowering in a greenhouse located at 56.229406N, 10.126424E, 8380, Trige, Denmark. FT 1st: weeks until first flowering period of the second year plants in 2015. FT 2nd: weeks until 2nd flowering time period of the second year plants in 2015. FT end: end of flowering of the second year plants in 2015. K^+ and Na^+: potassium and sodium ion accumulation in roots. ctrl: control. salt: salt treated. Weight, Size, Perimeter (Per), Length, Width and Circularity (Circ), refer to seed properties. Source data are provided as a Source Data file.

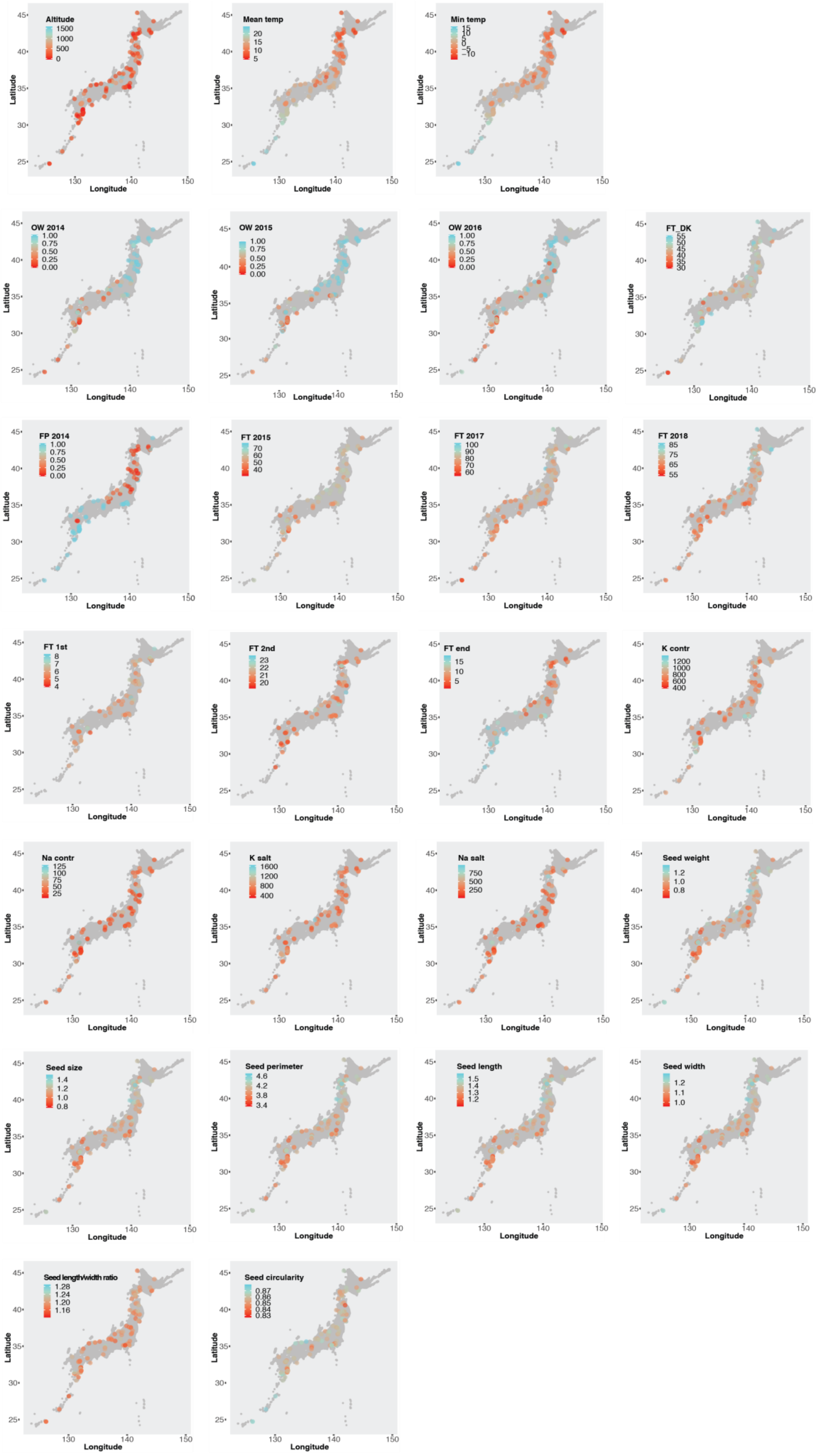
Altitude 1500 1000 500 0
Mean temp 20 15 10 5
Min temp
OW 2014 1.00 0.75 0.50 0.25 0.00
OW 2015 1.00 0.75 0.50 0.25 0.00
OW 2016 1.00 0.75 0.50 0.25 0.00
FT_DK 55 50 45 40 35 30
FP 2014 1.00 0.75 0.50 0.25 0.00
FT 2015 70 60 50 40
FT 2017 100 90 80 70 60
FT 2018 85 75 65 55
FT 1st 8 7 6 5 4
FT 2nd 23 22 21 20
FT end 15 10 5
K contr 1200 1000 800 600 400
Na contr 125 100 75 50 25
K salt 1600 1200 800 400
Na salt 750 500 250
Seed weight 1.2 1.0 0.8
Seed size 1.4 1.2 1.0 0.8
Seed perimeter 4.6 4.2 3.8 3.4
Seed length 1.5 1.4 1.3 1.2
Seed width 1.2 1.1 1.0
Seed length/width ratio 1.28 1.24 1.20 1.16
Seed circularity 0.87 0.86 0.85 0.84 0.83
Latitude
Longitude

Supplementary Figure 6. Phenotypes and geographic origin. See **Supplementary Data 1** for phenotype descriptions. Source data are provided as a Source Data file.

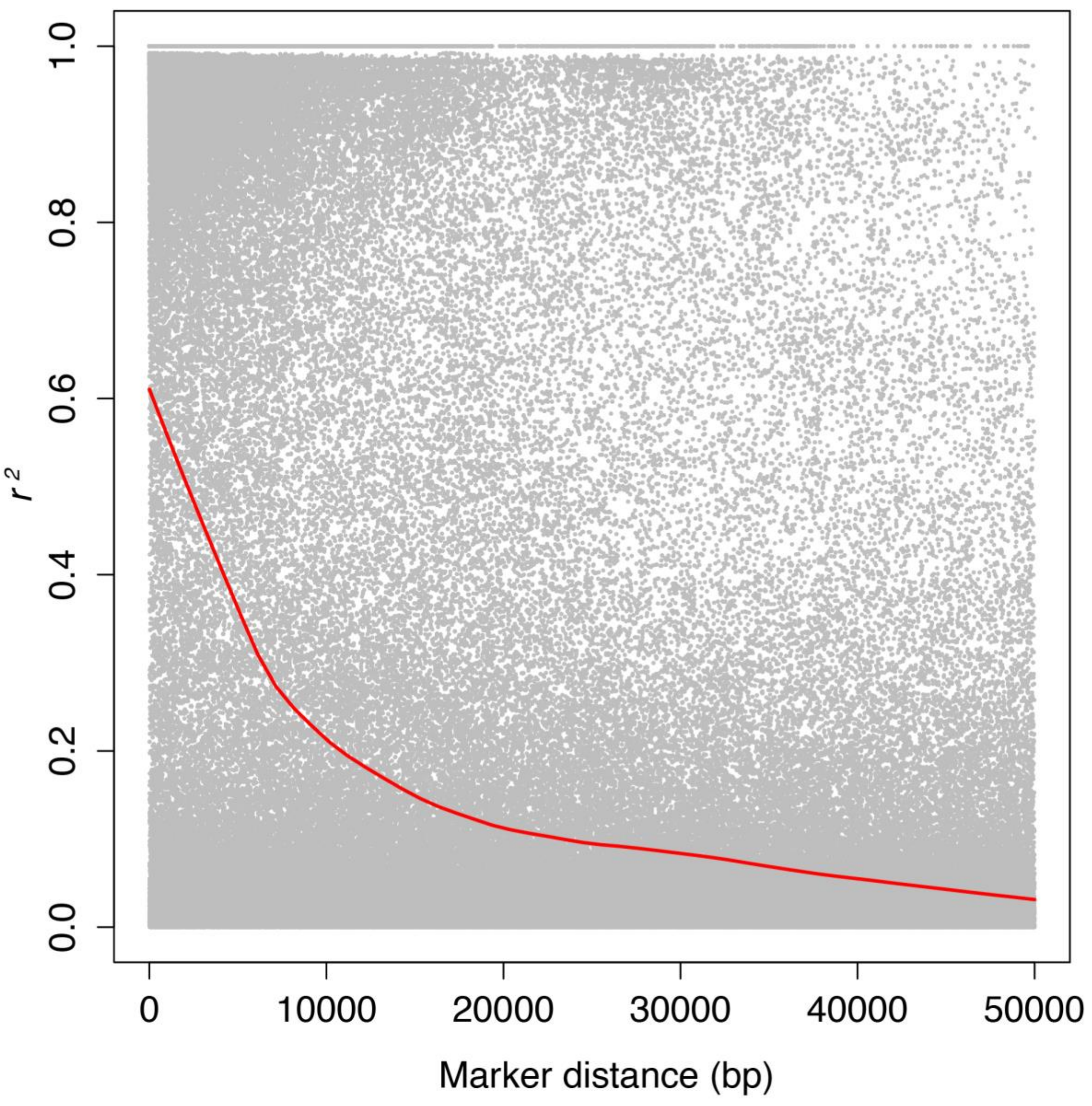

Supplementary Figure 7. LD decay. Linkage disequilibrium (r^2) as a function of pairwise marker distance.

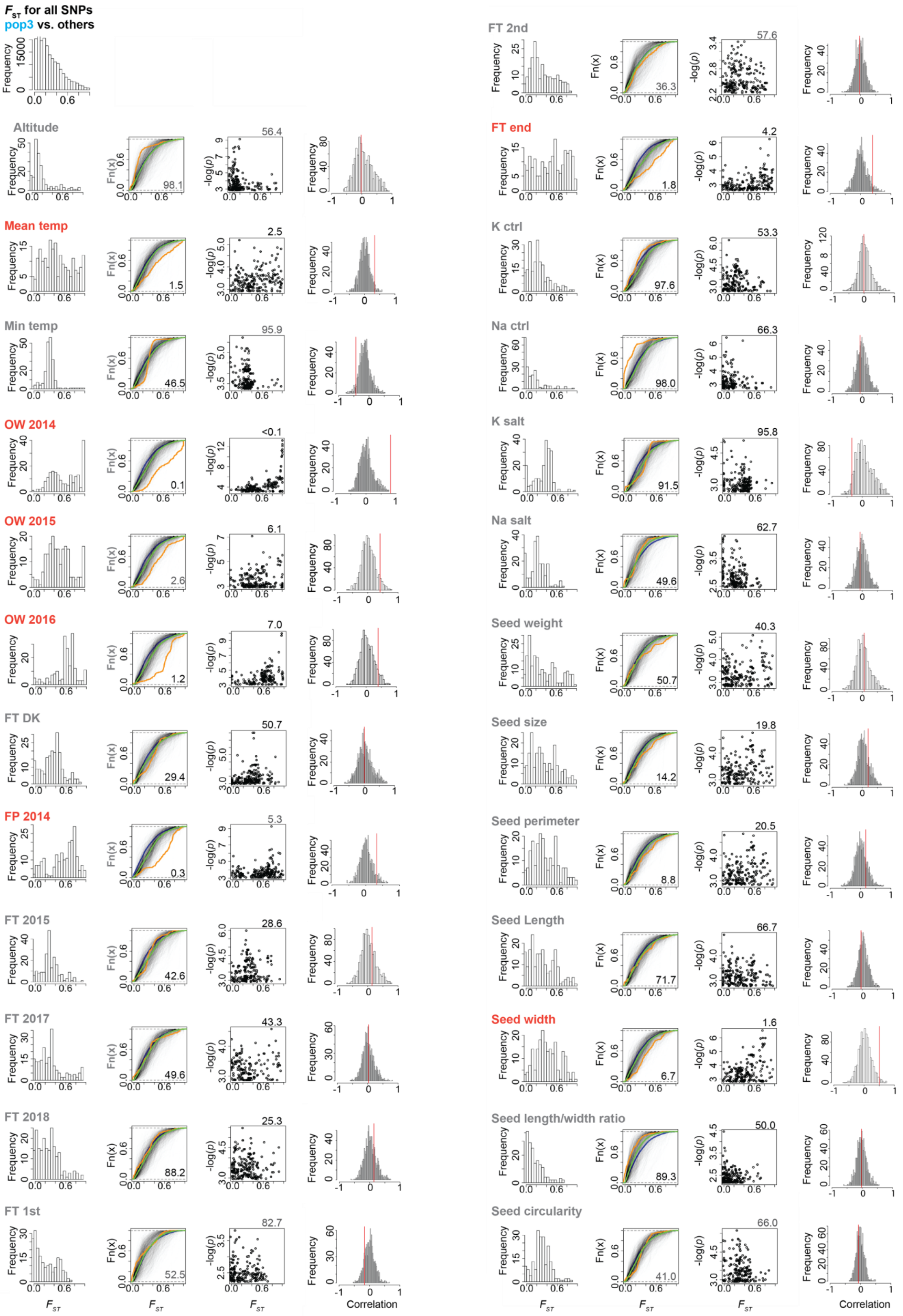

FST for all SNPs
pop3 vs. others
Altitude
Mean temp
Min temp
OW 2014
OW 2015
OW 2016
FT DK
FP 2014
FT 2015
FT 2017
FT 2018
FT 1st
FT 2nd
FT end
K ctrl
Na ctrl
K salt
Na salt
Seed weight
Seed size
Seed perimeter
Seed Length
Seed width
Seed length/width ratio
Seed circularity
Frequency
Fn(x)
-log(p)
FST
Correlation

Supplementary Figure 8. GWA and F_{ST} overlaps for pop3 vs non-pop3. The leftmost panel shows distributions of F_{ST} values for all SNPs or for the 200 SNPs with lowest -log(p) GWA scores for the traits indicated. The second panel from the left shows experimental cumulative distribution graphs for the F_{ST} values for all SNPs (blue) and the SNPs shown in the panel to the left (orange). If the orange curve lies below the blue curve, it indicates a shift to higher F_{ST} values for the SNPs with high GWA scores. Each of the semi-transparent black lines indicate F_{ST} distributions for the top 200 SNPs from a GWA analysis of permuted data. The green line indicates the distribution for all 1000 sets of permuted data. The false discovery rate shown in the lower right hand corner indicates the frequency (%) of permuted distributions shifted to higher F_{ST} values than the observed data. The third panel from the left shows F_{ST} values plotted versus GWA -log(p) scores. The false discovery rate shown in the upper right hand corner indicates the frequency (%) of F_{ST} versus GWA -log(p) Spearman correlation coefficients with larger values than the correlation coefficient of the observed data. The rightmost panel shows a histogram of F_{ST} versus GWA -log(p) Spearman correlation coefficients obtained from 1000 sets of permuted data. The red vertical line indicates the observed Spearman correlation coefficient. Altitude, Average (Avg) and Minimum (Min) temperature (temp) refer to accession collection sites. OW: overwintering, the fraction of plants surviving winter. FP 2014: Proportion of plants flowering at the Tohoku field site in 2014. FT: flowering time. FT DK: days to flowering in a greenhouse located at 56.229406N, 10.126424E, 8380, Trige, Denmark. FT 1st: weeks until first flowering period of the second year plants in 2015. FT 2nd: weeks until 2nd flowering time period of the second year plants in 2015. FT end: end of flowering of the second year plants in 2015. K^+ and Na^+: potassium and sodium ion accumulation in roots. ctrl: control. salt: salt treated. Traits likely associated with local adaptation are highlighted (orange). Source data are provided as a source data file.

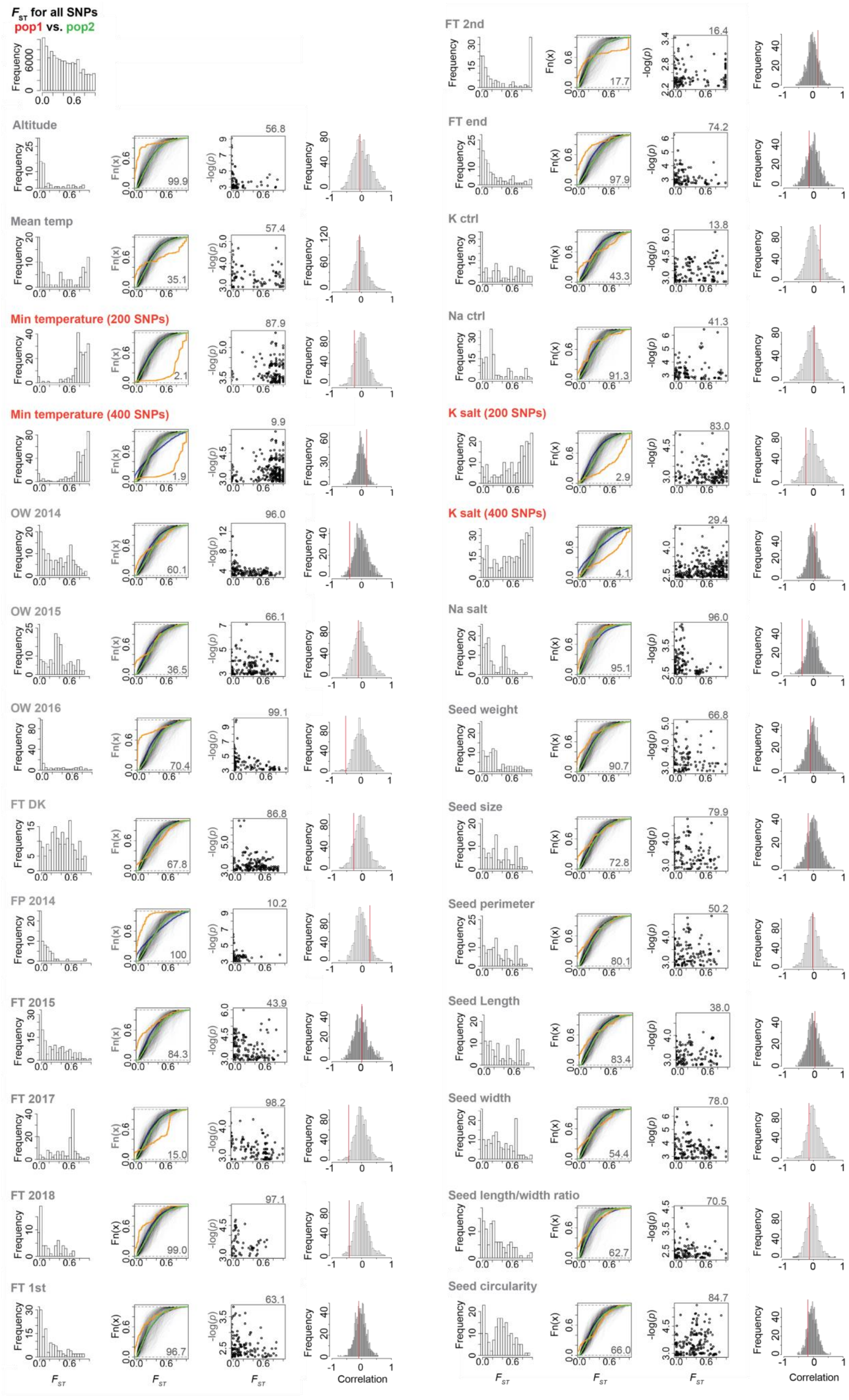
F_{ST} for all SNPs
pop1 vs. pop2
Altitude
Mean temp
Min temperature (200 SNPs)
Min temperature (400 SNPs)
OW 2014
OW 2015
OW 2016
FT DK
FP 2014
FT 2015
FT 2017
FT 2018
FT 1st
FT 2nd
FT end
K ctrl
Na ctrl
K salt (200 SNPs)
K salt (400 SNPs)
Na salt
Seed weight
Seed size
Seed perimeter
Seed Length
Seed width
Seed length/width ratio
Seed circularity
Frequency
Fn(x)
-log(p)
F_{ST}
Correlation

Supplementary Figure 9. GWA and F_{ST} overlaps for pop1 vs pop2 comparison. The leftmost panel shows distributions of F_{ST} values for all SNPs or for the 200 SNPs with lowest -log(*p*) GWA scores for the traits indicated. The second panel from the left shows experimental cumulative distribution graphs for the F_{ST} values for all SNPs (blue) and the SNPs shown in the panel to the left (orange). If the orange curve lies below the blue curve, it indicates a shift to higher F_{ST} values for the SNPs with high GWA scores. Each of the semi-transparent black lines indicate F_{ST} distributions for the top 200 SNPs from a GWA analysis of permuted data. The green line indicates the distribution for all 1000 sets of permuted data. The false discovery rate shown in the lower right hand corner indicates the frequency (%) of permuted distributions shifted to higher F_{ST} values than the observed data. The third panel from the left shows F_{ST} values plotted versus GWA -log(*p*) scores. The false discovery rate shown in the upper right hand corner indicates the frequency (%) of F_{ST} versus GWA -log(*p*) Spearman correlation coefficients with larger values than the correlation coefficient of the observed data. The rightmost panel shows a histogram of F_{ST} versus GWA -log(*p*) Spearman correlation coefficients obtained from 1000 sets of permuted data. The red vertical line indicates the observed Spearman correlation coefficient. Altitude, Average (Avg) and Minimum (Min) temperature (temp) refer to accession collection sites. OW: overwintering, the fraction of plants surviving winter. FP 2014: Proportion of plants flowering at the Tohoku field site in 2014. FT: flowering time. FT DK: days to flowering in a greenhouse located at 56.229406N, 10.126424E, 8380, Trige, Denmark. FT 1st: weeks until first flowering period of the second year plants in 2015. FT 2nd: weeks until 2nd flowering time period of the second year plants in 2015. FT end: end of flowering of the second year plants in 2015. K^+ and Na^+: potassium and sodium ion accumulation in roots. ctrl: control. salt: salt treated. Traits likely associated with local adaptation are highlighted (orange). All analyses were carried out using top 200 GWA SNPs, unless otherwise indicated. Source data are provided as a source data file.

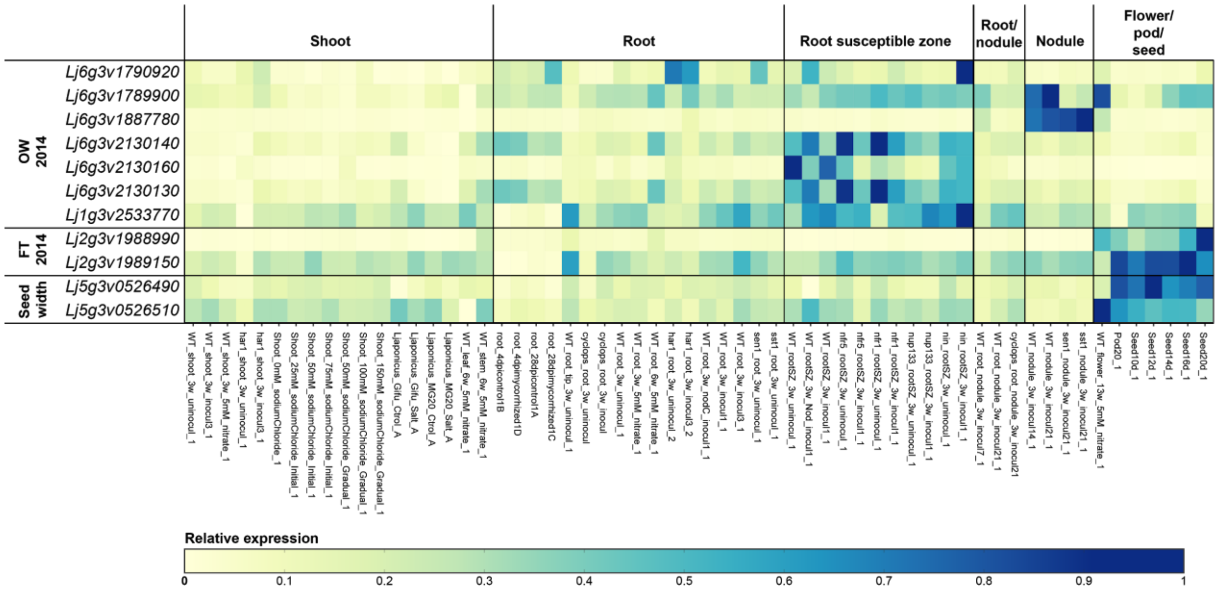

Supplementary Figure 10. Candidate gene expression patterns. The associated trait is indicated on the left. OW: overwintering. FT 2014: candidate genes associated with both flowering proportion 2014 and the end of flowering in 2015. Expression data was retrieved from Lotus Base (https://lotus.au.dk).

Lj6g3v1790920: RING-type zinc finger protein

Lj6g3v1789900: 1-aminocyclopropane-1-carboxylate oxidase

Lj6g3v1887780: HBS1-like protein, Alpha/Beta hydrolase fold (IPR029058)

Lj6g3v2130140: G-type lectin S-receptor-like serine/threonine-protein kinase

Lj6g3v2130160: G-type lectin S-receptor-like serine/threonine-protein kinase

Lj6g3v2130130: Glutamyl-tRNA(Gln) amidotransferase subunit A protein

Lj2g3v1988990: glutamate dehydrogenase-like

Lj2g3v1989150: *EMBRYONIC FLOWER 2*-like

Lj5g3v0526490: mitochondrial glycoprotein family protein

Lj5g3v0526510: putative thiol peptidase family protein

Supplementary Table 1. Survival rates and metadata.

Accession	pop1	pop2	pop3	Miyazaki Survival rate	Tohoku survival rate	Miyazaki - Tohoku rate	Latitude	Mean temp	Min temp
**MG030**	0.00	0.00	1.00	0.27	1.00	-0.73	42.85	6.4	-14.3
**MG007**	0.00	0.00	1.00	0.57	1.00	-0.43	39.32	8.7	-7.4
Gifu	0.00	0.36	0.64	0.88	0.96	-0.08	35.72	11.2	-5.5
MG076	0.06	0.28	0.66	0.67	0.64	0.03	34.26	12.6	-1.9
MG005	0.01	0.10	0.89	0.94	1.00	-0.06	35.3	15.4	0.1
**MG008**	0.00	1.00	0.00	0.63	0.82	-0.19	35.02	15.2	0.3
**MG066**	0.00	1.00	0.00	0.88	0.29	0.60	32.13	17.2	1.5
**MG020**	0.76	0.23	0.01	0.72	0.00	0.72	24.72	23.8	15.7

pop1-3: population membership. The accessions are sorted by minimum temperature (Min temp) at the site of origin. The "Miyazaki - Tohoku rate" column indicates the difference in survival rate between the Miyazaki and Tohoku sites.

Supplementary Table 2. Survival rates and *p*-values for all years.

	Miyazaki vs. Tohoku		**Miyazaki**	**Tohoku**
All years	*p*	*pop3 vs. non-pop3*	*0.0203*	*0.0414*
pop3	*6.19e-07*	survive	12	20
		die	17	0
		survival rate	41%	100% (87%)
Non-pop3	*1.76e-08*	survive	33	13
		die	10	36
		survival rate	77%	27% (36%)

All years: data from 2014, 2015 and 2016 was included in the analysis. Numbers in parentheses indicate the survival rates for all pop3 and non-pop3 individuals grown in all years. Non-italic numbers in the Miyazaki and Tohoku columns indicate numbers of individual plants surviving or dying from one year to the next. Numbers in italics indicate *p*-values (mixed linear model ANOVA) that pop3 (MG007 and MG030) vs. non-pop3 (MG008, MG020, MG066) individuals show the same performance at one location (Tohoku and Miyazaki columns) or that pop3 or non-pop3 individuals show the same performance at the two locations (Miyazaki vs. Tohoku column).

Supplementary Table 3. Survival rates and *p*-values for 2014.

	Miyazaki vs. Tohoku		**Miyazaki**	**Tohoku**
2014	*p*	*pop3 vs. non-pop3*	*0. 0203*	*0.0573*
pop3	*0.0013*	survive	12	9
		die	17	0
		survival rate	41%	100%
Non-pop3	*0.0010*	survive	33	7
		die	10	13
		survival rate	77%	35%

2014: data from 2014 was included in the analysis. Non-italic numbers in the Miyazaki and Tohoku columns indicate numbers of individual plants surviving or dying from one year to the next. Numbers in italics indicate *p*-values (mixed linear model ANOVA) that pop3 (MG007 and MG030) vs. non-pop3 (MG008, MG020, MG066) individuals show the same performance at one location (Tohoku and Miyazaki columns) or that pop3 or non-pop3 individuals show the same performance at the two locations (Miyazaki vs. Tohoku column).

Supplementary Table 4. Survival rates and *p*-values for 2014.

	Miyazaki vs. Tohoku		**Miyazaki**	**Tohoku**
2015	*p*	*pop3 vs. non-pop3*	*0. 0203*	*0.1720*
pop3	*0.0014*	survive	12	8
		die	17	0
		survival rate	41%	100%
Non-pop3	*0.0009*	survive	33	5
		die	10	11
		survival rate	77%	31%

2015: data from 2015 was included in the analysis. Non-italic numbers in the Miyazaki and Tohoku columns indicate numbers of individual plants surviving or dying from one year to the next. Numbers in italics indicate *p*-values (mixed linear model ANOVA) that pop3 (MG007 and MG030) vs. non-pop3 (MG008, MG020, MG066) individuals show the same performance at one location (Tohoku and Miyazaki columns) or that pop3 or non-pop3 individuals show the same performance at the two locations (Miyazaki vs. Tohoku column).

Supplementary Table 5. Survival rates and *p*-values for 2014.

	Miyazaki vs. Tohoku		**Miyazaki**	**Tohoku**
2016	*p*	*pop3 vs. non-pop3*	*0. 0203*	*0.0002*
pop3	*0.0397*	survive	12	3
		die	17	0
		survival rate	41%	100%
Non-pop3	*4.56e-08*	survive	33	1
		die	10	12
		survival rate	77%	8%

2016: data from 2016 was included in the analysis. Non-italic numbers in the Miyazaki and Tohoku columns indicate numbers of individual plants surviving or dying from one year to the next. Numbers in italics indicate *p*-values (mixed linear model ANOVA) that pop3 (MG007 and MG030) vs. non-pop3 (MG008, MG020, MG066) individuals show the same performance at one location (Tohoku and Miyazaki columns) or that pop3 or non-pop3 individuals show the same performance at the two locations (Miyazaki vs. Tohoku column).
