# Supplementary material for: Extreme genetic signatures of local adaptation during Lotus japonicus colonization of Japan
Source: Nat Commun. 2020 Jan 14;11:253. doi: 10.1038/s41467-019-14213-y (PMC6959357; doi:10.1038/s41467-019-14213-y)
Supplement: Supplementary file 3 — Reporting Summary [file 41467_2019_14213_MOESM3_ESM.pdf]

nature research

Corresponding author(s): Stig U. Andersen
Mikkel Heide Schierup
Shusei Sato

Last updated by author(s): Dec 18, 2019

Reporting Summary

Nature Research wishes to improve the reproducibility of the work that we publish. This form provides structure for consistency and transparency in reporting. For further information on Nature Research policies, see Authors & Referees and the Editorial Policy Checklist.

Statistics

For all statistical analyses, confirm that the following items are present in the figure legend, table legend, main text, or Methods section.

n/a	Confirmed	
☐	☒	The exact sample size (n) for each experimental group/condition, given as a discrete number and unit of measurement
☒	☐	A statement on whether measurements were taken from distinct samples or whether the same sample was measured repeatedly
☐	☒	The statistical test(s) used AND whether they are one- or two-sided<br>*Only common tests should be described solely by name; describe more complex techniques in the Methods section.*
☒	☐	A description of all covariates tested
☐	☒	A description of any assumptions or corrections, such as tests of normality and adjustment for multiple comparisons
☐	☒	A full description of the statistical parameters including central tendency (e.g. means) or other basic estimates (e.g. regression coefficient) AND variation (e.g. standard deviation) or associated estimates of uncertainty (e.g. confidence intervals)
☐	☒	For null hypothesis testing, the test statistic (e.g. F, t, r) with confidence intervals, effect sizes, degrees of freedom and P value noted<br>*Give P values as exact values whenever suitable.*
☒	☐	For Bayesian analysis, information on the choice of priors and Markov chain Monte Carlo settings
☒	☐	For hierarchical and complex designs, identification of the appropriate level for tests and full reporting of outcomes
☒	☐	Estimates of effect sizes (e.g. Cohen's d, Pearson's r), indicating how they were calculated

Our web collection on statistics for biologists contains articles on many of the points above.

Software and code

Policy information about availability of computer code

Data collection	SmartGrain (version number is not indicated, only a single version is available)
Data analysis	R v. 3.4.3 and 3.5.1, R-packages: ggplot v. 3.1.1, maps v. 3.3.0, mapdata v. 2.3.0, plotly v. 4.8.0, reshape2 v. 1.4.3, corrgram v. 1.13, lme4 v. 1.1-21, lmerTest v. 3.1-0, multcomp v. 1.4-10, qqman v. 0.1.4, zoo v. 1.8-5, viridis v. 0.5.1, grid v. 3.5.1, dplyr v. 0.8.0.1, gridExtra v. 2.3, adegenet v. 2.1.1, hierfstat v. 0.04-22, and pegas v. 0.11<br>Beagle version 5.0<br>Burrows-Wheeler Aligner (BWA) mem v. 0.75a<br>Picard v. 1.96.<br>Genome Analysis Tool Kit (GATK) v. 2.7-2 pipeline<br>VCF tools version 0.1.9<br>EIGENSOFT v. 6.0beta<br>VCFtools v. 0.1.9<br>fastSTRUCTURE version 1.0<br>SAMtools v1.3<br>BCFtools v1.3<br>EMMA (https://app.assembla.com/spaces/atgwas/git/source)<br>GCTA-GREML 1.91.7beta<br>Permutations using (https://github.com/bvilhjal/mixmogam/blob/lotus/examples.py)<br>Custom scripts and workflows are freely available on GitHub in the repositories https://github.com/ShahNiraj/JapanHistory, https://github.com/cks2903/Lotus_data_2019, and https://github.com/bvilhjal/mixmogam/blob/lotus/examples.py.

For manuscripts utilizing custom algorithms or software that are central to the research but not yet described in published literature, software must be made available to editors/reviewers. We strongly encourage code deposition in a community repository (e.g. GitHub). See the Nature Research guidelines for submitting code & software for further information.

Data

Policy information about availability of data

All manuscripts must include a data availability statement. This statement should provide the following information, where applicable:

- Accession codes, unique identifiers, or web links for publicly available datasets
- A list of figures that have associated raw data
- A description of any restrictions on data availability

Accessions read data have been deposited in the European Nucleotide Archive (ENA) database with accession number PRJEB27969[https://www.ebi.ac.uk/ena/data/view/PRJEB27969]. Genotype data is available for online GWA analysis through a website (https://lotus.au.dk/gwas/) based on the GWAPP platform 36, where it can be selected during the Genotype step when creating a new GWAS analysis. The phenotype data and GWA analyses are also available from the site (https://lotus.au.dk/gwas/#/study/68/overview). Data supporting the findings of this work are available within the paper and its Supplementary Information files. A reporting summary for this Article is available as a Supplementary Information file. The datasets generated and analyzed during the current study are available from the corresponding author upon request. The source data underlying Figures 1d-e, 4a, c, 5b and 6, and Supplementary figures 1, 5, 6, 8 and 9 are provided as a Source Data file.

Field-specific reporting

Please select the one below that is the best fit for your research. If you are not sure, read the appropriate sections before making your selection.

☒ Life sciences ☐ Behavioural & social sciences ☐ Ecological, evolutionary & environmental sciences

For a reference copy of the document with all sections, see nature.com/documents/nr-reporting-summary-flat.pdf

Life sciences study design

All studies must disclose on these points even when the disclosure is negative.

Sample size	A sample size of 136 wild Lotus japonicus accessions was chosen because this represented the entire collection at the time of the study.
Data exclusions	No data was excluded from the analysis.
Replication	The experiments were repeated across multiple years at same field site. As described in the manuscript, results varied between years due to different planting dates and climatic conditions.
Randomization	Randomization was not used to avoid placing many accessions originating from the same region next to each other by chance. Instead, the accessions were subdivided into three groups, each covering a wide range of geographic origins. Within each of these groups, the accessions were planted ordered by their accession ID in the column direction in the field.
Blinding	Blinding was not relevant, since group allocation was not used. All accessions were individually assessed and the phenotype data was then used in genome-wide association studies.

Reporting for specific materials, systems and methods

We require information from authors about some types of materials, experimental systems and methods used in many studies. Here, indicate whether each material, system or method listed is relevant to your study. If you are not sure if a list item applies to your research, read the appropriate section before selecting a response.

Materials & experimental systems

n/a	Involved in the study	
☒	☐	Antibodies
☒	☐	Eukaryotic cell lines
☒	☐	Palaeontology
☒	☐	Animals and other organisms
☒	☐	Human research participants
☒	☐	Clinical data

Methods

n/a	Involved in the study	
☒	☐	ChIP-seq
☒	☐	Flow cytometry
☒	☐	MRI-based neuroimaging
