# Supplementary material for: Extreme genetic signatures of local adaptation during Lotus japonicus colonization of Japan
Source: Nat Commun. 2020 Jan 14;11:253. doi: 10.1038/s41467-019-14213-y (PMC6959357; doi:10.1038/s41467-019-14213-y)
Supplement: Supplementary file 4 — Description of Additional Supplementary Files [file 41467_2019_14213_MOESM4_ESM.docx]

**Description of Additional Supplementary Files**

File Name: Supplementary Data 1
Description: Accession metadata. Read mapping and variant call statistics, geographic origin, population membership and phenotypes are described for each accession.

File Name: Supplementary Data 2
Description: GWA and *F*_ST_ (pop1 vs. pop2) overlaps for all traits and chromosomes. *F*_ST_ averages for 10 SNPs are indicated by grey dots. In the left panel, colored dots indicate *F*_ST_ values for SNPs with GWA -log(*p*) scores > 3 for the chromosomes and traits indicated. In the right panel *F*_ST_ averages for 10 SNPs (grey dots) are overlaid with GWAS -log(*p*) scores > 3 for the chromosomes and traits indicated. Each chromosome is shown on a separate page. Viewing at 500% is recommended.

File Name: Supplementary Data 3
Description: GWA and *F_ST_* (pop3 vs. non-pop3) overlaps for all traits and chromosomes. *F*_ST_ averages for 10 SNPs are indicated by grey dots. In the left panel, colored dots indicate *F*_ST_ values for SNPs with GWA -log(*p*) scores > 3 for the chromosomes and traits indicated. In the right panel *F*_ST_ averages for 10 SNPs (grey dots) are overlaid with GWAS -log(*p*) scores > 3 for the chromosomes and traits indicated. Each chromosome is shown on a separate page. Viewing at 500% is recommended.

File Name: Supplementary Data 4
Description: Validated SNPs. List of validated SNP, their validated MG20 and Gifu genotypes, and the genotypes called based on read mapping data at the same sites are listed.
