# Supplementary figures and images for: Extreme genetic signatures of local adaptation during Lotus japonicus colonization of Japan
Source: Nat Commun. 2020 Jan 14;11:253. doi: 10.1038/s41467-019-14213-y (PMC6959357; doi:10.1038/s41467-019-14213-y)

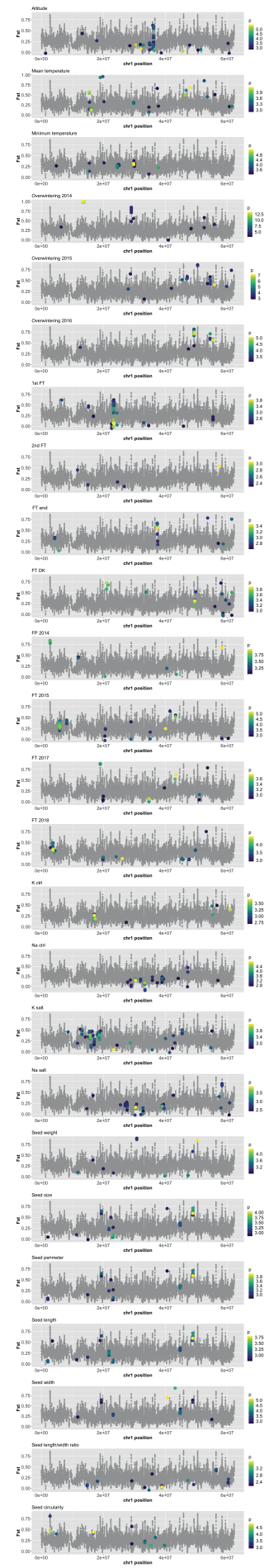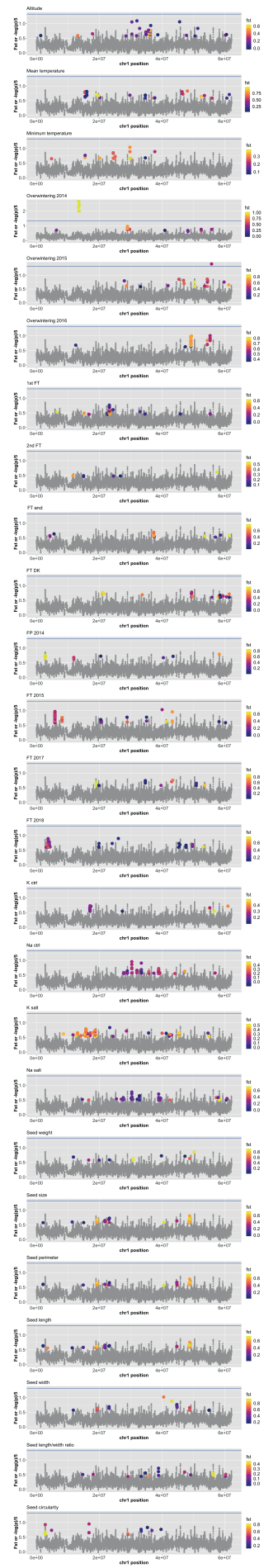

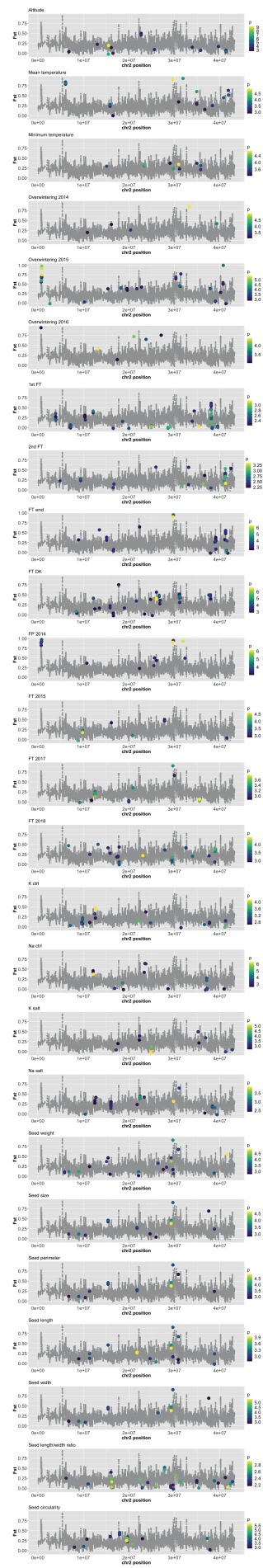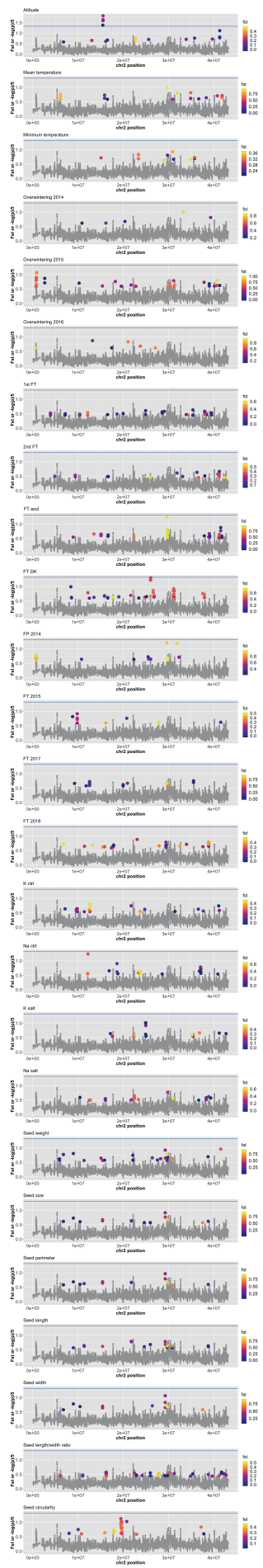

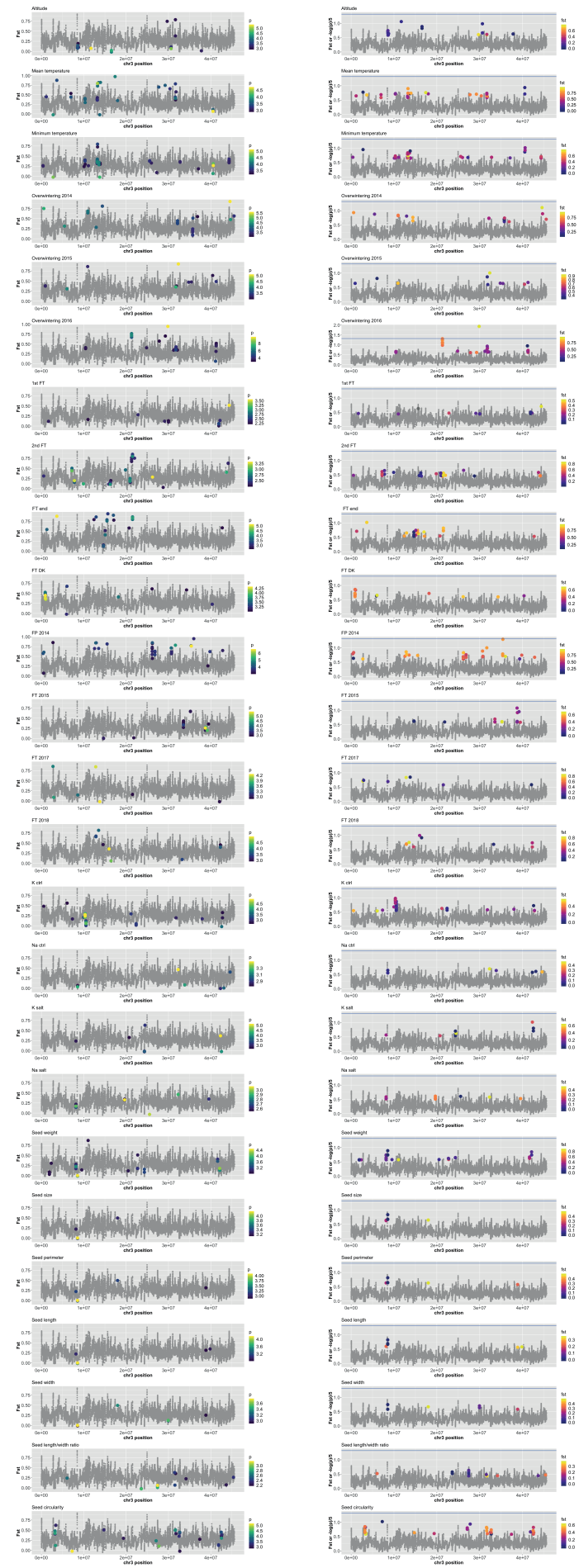

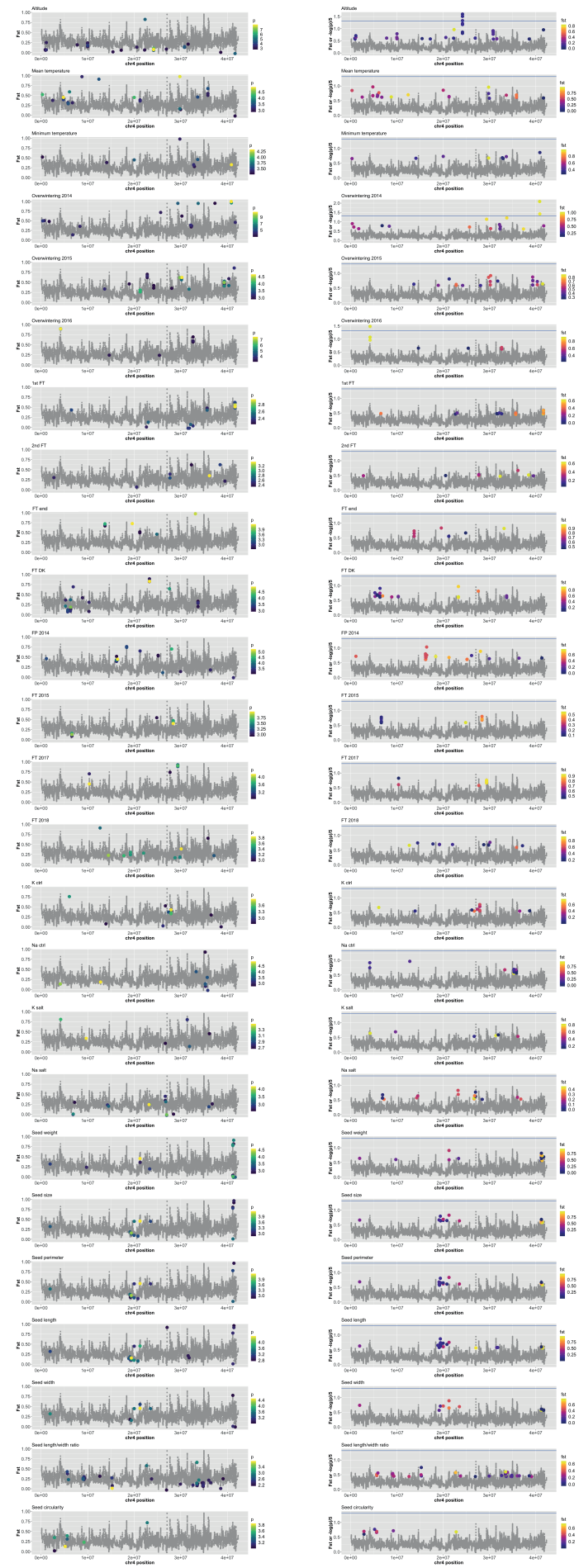

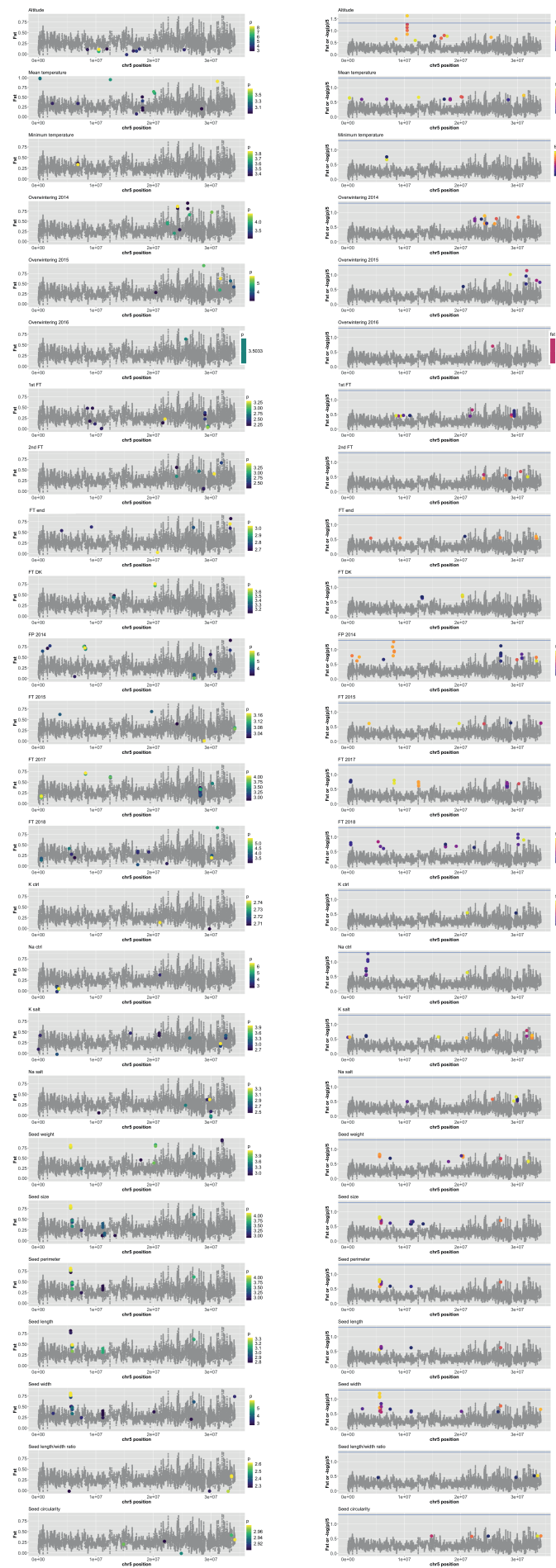

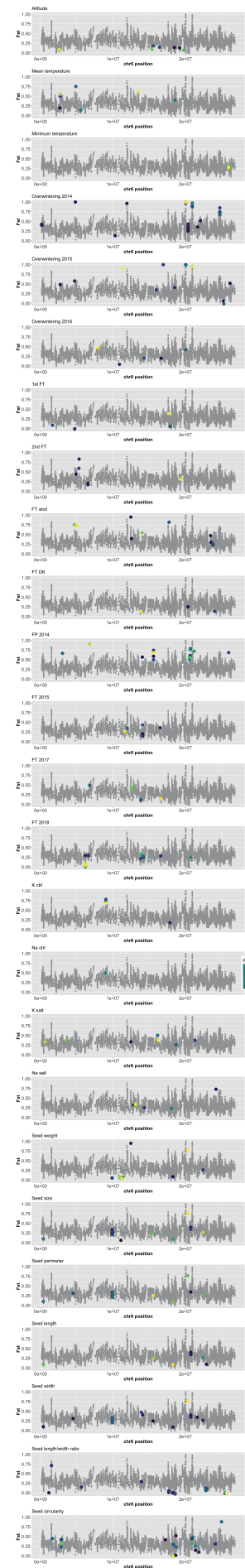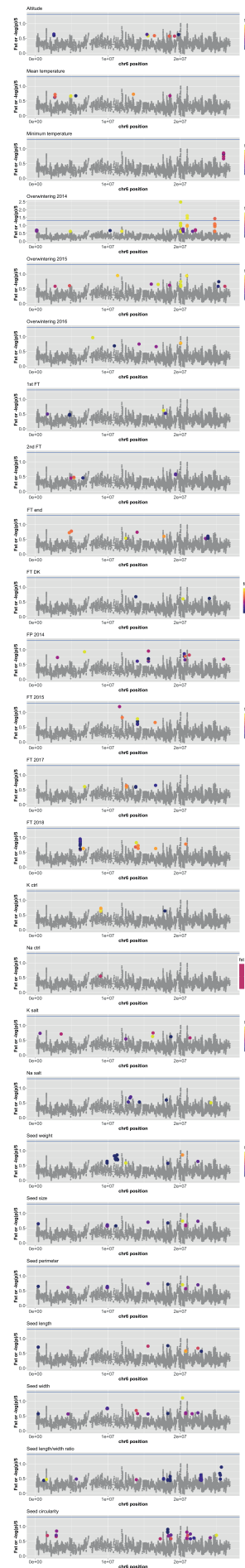

Supplement: Supplementary file 6 — Supplementary Data 2 [file 41467_2019_14213_MOESM6_ESM.pdf]

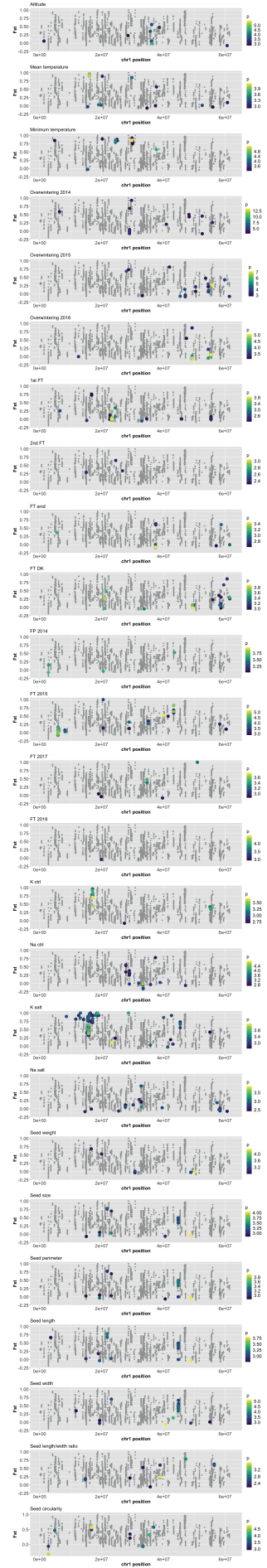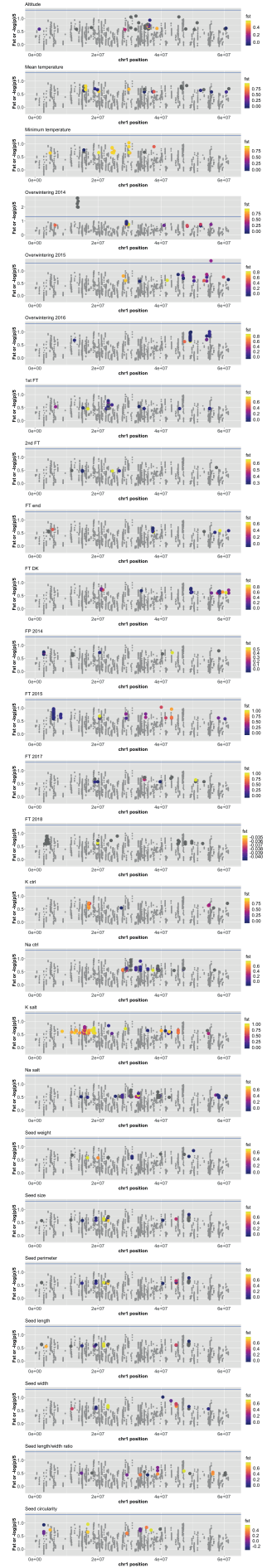

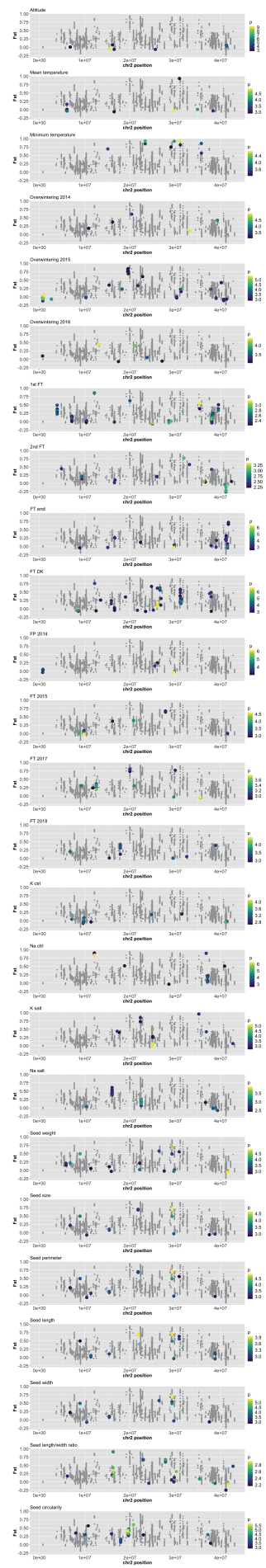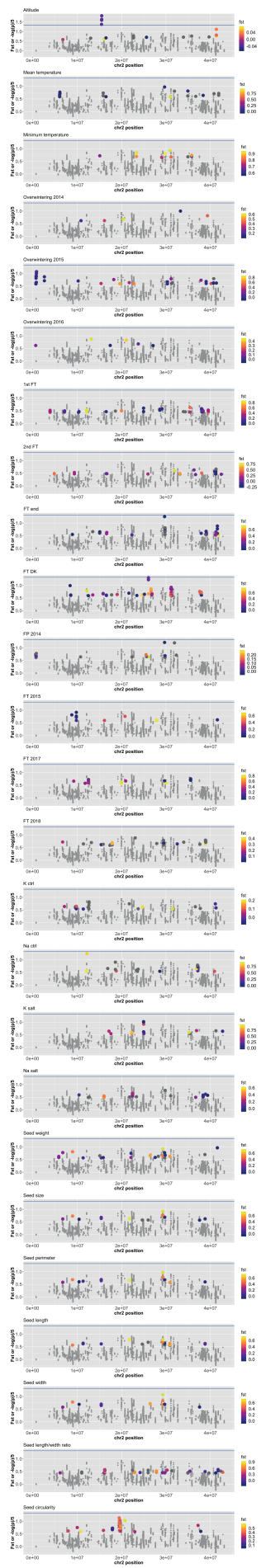

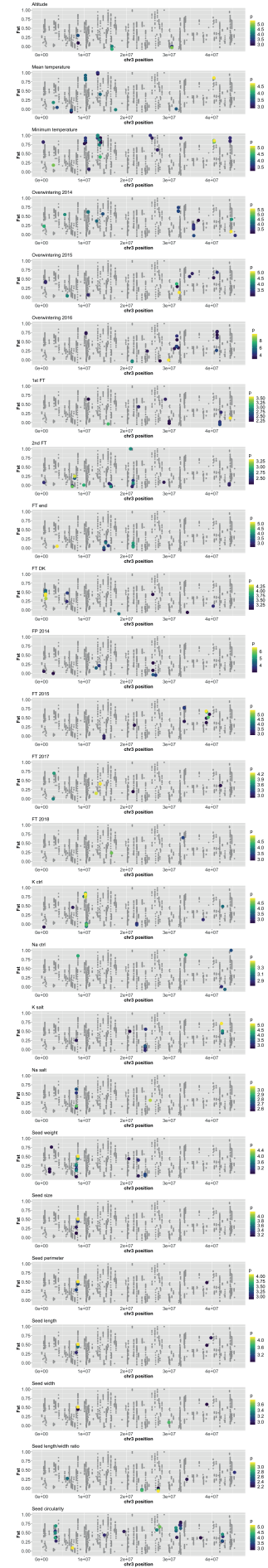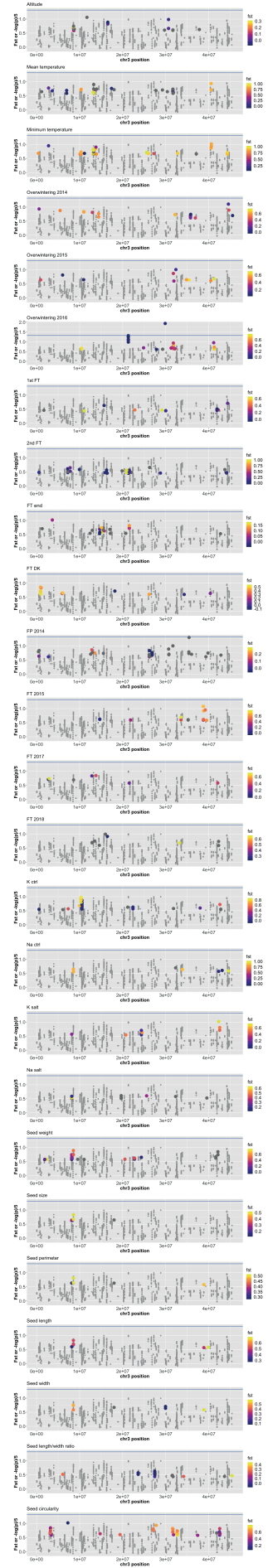

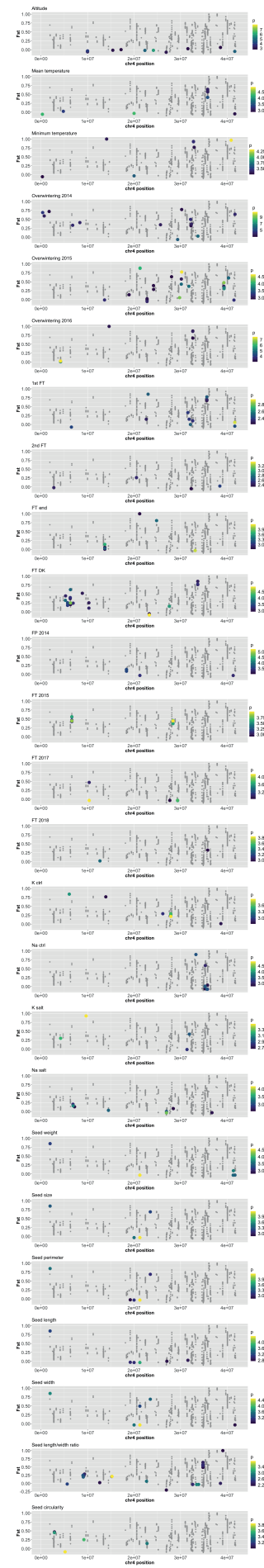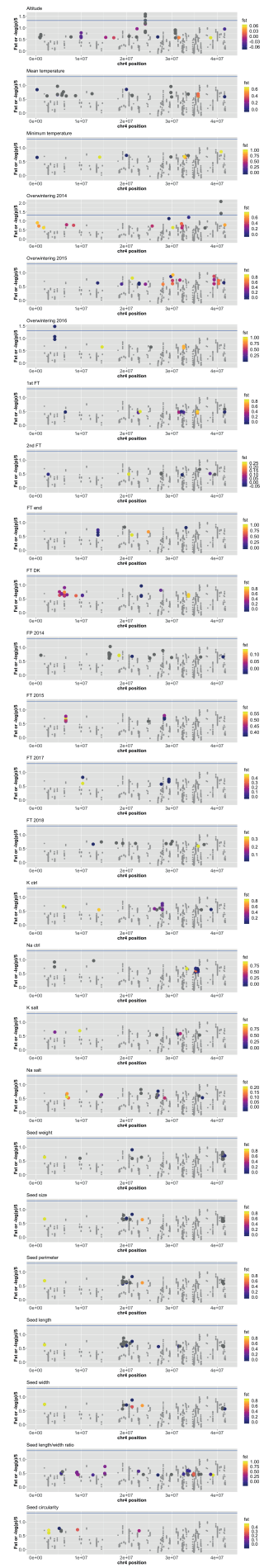



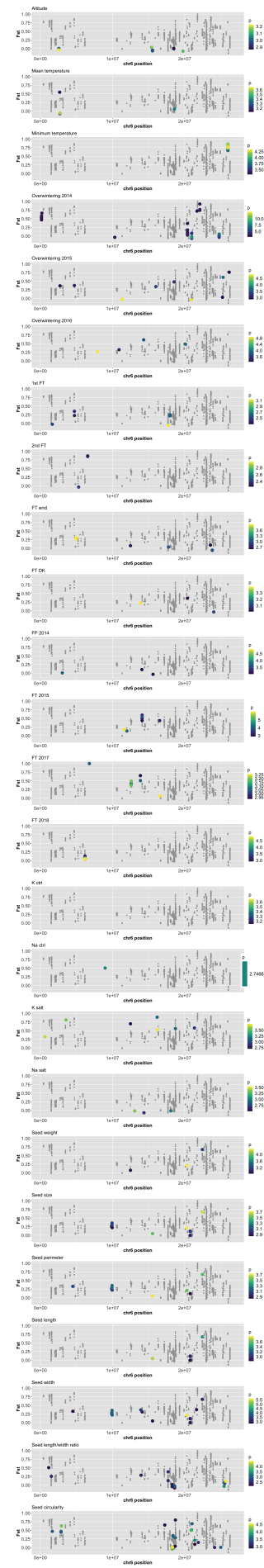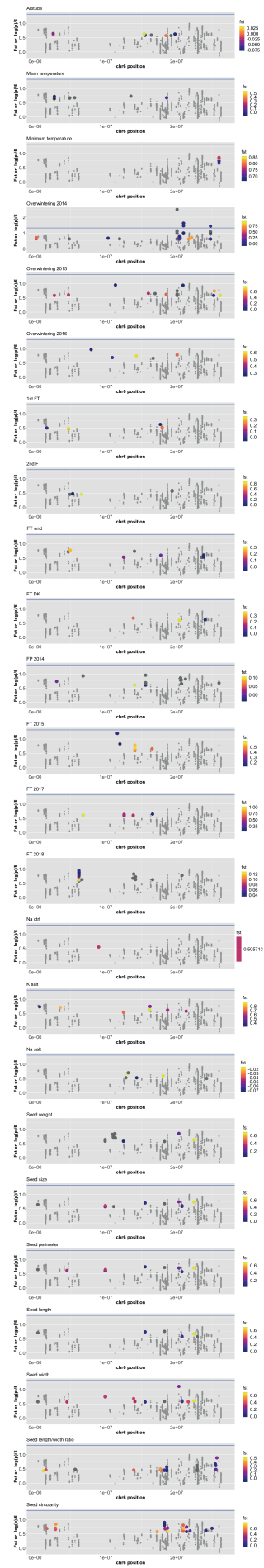

Supplement: Supplementary file 7 — Supplementary Data 3 [file 41467_2019_14213_MOESM7_ESM.pdf]
